# Diagnostic performance of quantitative coronary artery disease assessment using computed tomography in patients with aortic stenosis undergoing transcatheter aortic-valve implantation

**DOI:** 10.1186/s12872-022-02623-8

**Published:** 2022-04-18

**Authors:** Daniel Malebranche, Maximilian K. M. Hoffner, Adrian T. Huber, Aleksandar Cicovic, Giancarlo Spano, Benedikt Bernhard, Joanna Bartkowiak, Taishi Okuno, Jonas Lanz, Lorenz Räber, Fabien Praz, Stefan Stortecky, Stephan Windecker, Thomas Pilgrim, Christoph Gräni

**Affiliations:** 1 Department of Cardiology, Inselspital, Bern University Hospital, University of Bern, 3010 Bern, Switzerland; 2 Department of Diagnostic, Interventional and Pediatric Radiology, Inselspital, University of Bern, Bern, Switzerland

**Keywords:** Transcatheter aortic valve replacement, Computed tomography angiography, CTA, TAVI, CAD, Coronary artery disease, QCA, Invasive angiography

## Abstract

**Background:**

Computed tomography angiography (CTA) is a cornerstone in the pre- transcatheter aortic valve replacement (TAVI) assessment. We evaluated the diagnostic performance of CTA and coronary artery calcium score (CACS) for CAD evaluation compared to invasive coronary angiography in a cohort of TAVI patients.

**Methods:**

In consecutive TAVI patients without prior coronary revascularization and device implants, CAD was assessment by quantitative analysis in CTA. (a) Patients with non-evaluable segments were classified as obstructive CAD. (b) In patients with non-evaluable segments a CACS cut-off of 100 was applied for obstructive CAD. The reference standard was quantitative invasive coronary angiography (QCA, i.e. ≥ 50% stenosis).

**Results:**

100 consecutive patients were retrospectively included, age was 82.3 ± 6.5 years and 30% of patients had CAD. In 16% of the patients, adequate visualization of the entire coronary tree (all 16 segments) was possible with CTA, while 84% had at least one segment which was not evaluable for CAD analysis due to impaired image quality. On a per-patient analysis, where patients with low image quality were classified as CAD, CTA showed a sensitivity of 100% (95% CI 88.4–100.0), specificity of 11.4% (95% CI 5.1–21.3), PPV of 32.6% (95% CI 30.8–34.5), NPV of 100% and diagnostic accuracy of 38% (95% CI 28.5–48.3) for obstructive CAD. When applying a combined approach of CTA (in patients with good image quality) and CACS (in patients with low image quality), the sensitivity and NPV remained at 100% and obstructive CAD could be ruled out in 20% of the TAVI patients, versus 8% using CTA alone.

**Conclusion:**

In routinely acquired pre-TAVI CTA, the image quality was insufficient in a high proportion of patients for the assessment of the entire coronary artery tree. However, when adding CACS in patients with low image quality to quantitative CTA assessment in patients with good image quality, obstructive CAD could be ruled-out in 1/5 of the patients and may therefore constitute a strategy to streamline pre-procedural workup, and reduce risk, radiation and costs in selected TAVI patients without prior coronary revascularization or device implants.

**Supplementary Information:**

The online version contains supplementary material available at 10.1186/s12872-022-02623-8.

## Background

Computed tomography angiography (CTA) is an essential component of the pre-interventional transcatheter aortic valve replacement (TAVI) assessment [1]. It provides detailed cross-sectional information of the aortic root including the aortic valvular apparatus and ascending aorta as well as the peripheral vessels. The use of this modality has been found to improve procedural outcomes [2–4].

All patients undergoing TAVI require pre-procedural assessment of coronary anatomy [5]. Invasive coronary angiography (ICA) has been considered the gold standard for assessment of coronary artery disease (CAD) in these patients with rather high pre-test probability [6]. Traditionally, CTA has had limited use in the clarification of coronary anatomy in patients with severe symptomatic aortic stenosis because the medications that facilitate visualization of the coronary tree, namely beta blockers and nitroglycerine, are frequently not well tolerated by patients with preload dependence and fixed afterload.

As the indication for TAVI expands to lower risk patients with modest pre-test probabilities for obstructive CAD, the added benefit of an invasive test that is associated with incremental risk, radiation and cost may be of limited value with important implications to both patients and healthcare systems [7]. Whether CTA may serve in this clinical setting in the future, as a rule-out test for CAD is unclear. The diagnostic accuracy of pre-procedural CTA in TAVI patients as compared to ICA with regard to CAD has previously been investigated, with conflicting findings [8–15]. This may be partly attributable to the use of visual (instead of quantitative) assessment as a means of grading the stenosis on both CTA and ICA. Furthermore, previous studies have not systematically included the Coronary artery calcium score (CACS) as part of the CT assessment for CAD.

The aim of the current investigation therefore, was to clarify the value of pre-TAVI CTA in delineating coronary anatomy by assessing its diagnostic performance (using both quantitative assessment of CTA and CACS and its combination) as compared to invasive quantitative coronary angiography (QCA) in a prospective cohort of consecutive TAVI patients. We hypothesize that the diagnostic performance of combined quantitative CTA/CACS assessment is such that CAD can be safely and reliably ruled out in select TAVI patients and ultimately obviate the need for additional evaluation with invasive coronary angiography.

## Methods

### Study population

All patients undergoing TAVI at Bern University Hospital, Bern, Switzerland, are consecutively enrolled into a prospective institutional registry that is a part of the Swiss TAVI registry (NCT01368250) [16]. The registry was approved by the local ethics committee (Kantonale Ethikkomission Bern, Switzerland), and patients provided written informed consent to participate. We are confirming that all experiments were performed in accordance with relevant guidelines and regulations. For the purpose of the present study, the sample size (n = 100) was aligned to previous studies with comparable hypothesis and baseline characteristics [8–15, 17, 18]. Consecutive patients with symptomatic, severe aortic stenosis undergoing CT angiography as part of the routine, pre-procedural TAVI work-up starting from 06/2018 were enrolled. Exclusion criteria were prior percutaneous coronary intervention or coronary artery bypass grafting, prior pacemaker implantation or valve in valve intervention. Furthermore, patients where CTAs were performed in external institutions were excluded. One independent blinded reader assessed CTA with regard to the coronary arteries and one independent blinded reader performed QCA.

### CT acquisition

Pre-TAVI CT was performed as previously published [19]. A standardized native CT scan of the entire heart for the CACS was routinely performed, followed by the electrocardiogram-gated multi-slice CT on a Siemens Somatom Definition Flash Dual-Source scanner with a slice collimation of 128 × 0.6 mm, tube voltage of 100 or 120 kV, and tube current according to patient size (Siemens Medical Solutions, Inc., Forchheim, Germany). Each patient received an intravenous injection of 80–120 mL of contrast medium at a flow rate of 4 mL/s and image acquisition was performed during an inspiratory breath-hold in a cranio-caudal direction. As per our institutional protocol all TAVI-CT scans were performed without the use of beta-blocker or nitroglycerin prior to CTA scanning. Acquired CT images were then transferred to a dedicated workstation.

### CT data assessment

A blinded experienced reader (MH), unaware of the clinical history and results from the invasive measurements, retrospectively performed quantitative CTA data analysis of the coronary arteries on a dedicated software (Syngovia, CT Coronary, Siemens Healthineers) after selecting the optimal reconstructed phase with regard to image quality. Coronary arteries were assessed according to the AHA segments [20] and each coronary artery segment larger than 1.5 mm in diameter was evaluated. As a first step, coronary artery segments were judged as either evaluable or non-evaluable, and the cross-sectional area of evaluable segments was measured. The lumen borders were generated by a dedicated software and manually edited as needed. To quantify the degree of stenosis, the smallest diameter of the cross-sectional area at the level of the lesion was compared to the smallest diameter of the disease-free segments immediately distal to the lesion. The percentage of stenosis was derived according to the formula: ((reference diameter-minimum diameter)/reference-minimum diameter) × 100.

The CACS from each coronary artery was obtained using the Agatston method [21] and summed using native CT images obtained from the pre-interventional CT using dedicated workstations (i.E. Syngo.via CaScore Siemens Healthineers, Forchheim, Germany).

### Invasive coronary angiography assessment

As in the case of the CT assessment, an experienced blinded reader (AC) analyzed coronary angiograms without the knowledge of clinical history or results from CT angiography. Coronary arteries were assessed according to the same segmented model used for the CT analysis [20]. Quantitative coronary angiography (QCA) was performed using QAngio XA 3D (Medis Medical Imaging, Schuttersveld, Leiden, The Netherlands). Identical to the CT analysis, the degree of stenosis was assessed measuring the smallest diameter of the cross-sectional area at the level of the lesion, and was compared to the smallest diameter of the disease-free segments immediately distal to the lesion. The percentage of stenosis was derived according to the same formula as for the CT analysis: ((reference minimum diameter - stenosis minimum diameter)/(reference minimum diameter)) × 100.

## Statistics

Patient information and results are reported for the entire cohort and grouped according to the presence or absence of obstructive CAD. Data are reported as median ± IQR from 25 to 75th percentile, mean ± SD or percentages, as appropriate. Continuous variables were analysed using the Student *t* test or Mann–Whitney U test, as appropriate. Categorical data were analysed with χ^2^ test or Fisher’s exact test. The *p* values of all outcomes were two-sided and values less than 0.05 were considered as statistically significant. CI was defined as 95%.

The pre-specified diagnostic accuracy was performed on a per-patient level with the reference standard of CAD as ≥ 50% and ≥ 70% stenosis on invasive QCA in one of the major epicardial coronary arteries according to the AHA-segment model. Furthermore, using the same cut-off in QCA was used on a per vessel level and on a per segment and patient level. In the first analysis, diagnostic accuracy of CTA was determined on an intention-to-diagnose basis, meaning that no coronary segment was excluded; non-evaluable segments were rated as stenosed, as previously reported [17]. In a separate analysis, we combined CTA results only from patients in whom all coronary segments were evaluable and different CACS thresholds were applied to those patients with non-evaluable segments in CTA. In patients with completely evaluable segments on CTA, the results from CACS were not included. The diagnostic accuracy and rule-out performance of CAD (sensitivity, specificity, positive predictive value and negative predictive value) in CTA/CACS as compared to the gold standard invasive QCA was determined on a per-patient and per-vessel/segment level. Receiver-operating characteristics (ROC) analysis was carried out to further assess the performance of CACS using ≥ 50% and ≥ 70% stenosis on invasive QCA as the reference standard. All statistical analyses were performed using IBM SPSS Statistics for Windows, V.25 (IBM Corporation, Armonk, New York, USA).

## Results

Between 06/2018 and 02/2019, 100 consecutive patients with severe aortic stenosis undergoing pre-TAVI CT at the Swiss Cardiovascular Center, Bern University Hospital, who fulfill the inclusion and exclusion criteria were analyzed for the present study. In detail, of the 211 patients with severe aortic stenosis who underwent a pre-TAVI CT, 45 patients with prior percutaneous coronary interventions with stent implantation, 15 patients with coronary artery bypass grafting, 7 patients with valve- in valve procedure, 5 patients with pacemakers, and 39 patients with external CTAs were excluded, resulting in 100 patients for the purpose of the present analysis.

### Baseline clinical characteristics

The baseline clinical characteristics of the cohort are shown in Table 1. The average age of patients was 82.3 ± 6.5 years, 70% were female and the mean Euro SCORE II Value of 4.9 ± 6.1. 30 patients had CAD and thereof 16 patients with 1-vessel-disease, 13 with 2-vessel-disease and 1 patient with 3-vessel-disease. TAVI patients with CAD had significantly higher CACS as compared with those with no CAD (1019 [542–1907] versus 386 [75–765]; *p* < 0.001).

**Table 1:** Baseline characteristics

	Total/mean ± SD	Invasive coronary angiography (QCA)		*p* value
		No obstructive CAD	Obstructive CAD	
Number of patients	100	70	30	
Female gender	70	50 (71.4%)	20 (66.7%)	0.812
BMI (kg/m^2^)	25.5 ± 5.6	24.9 ± 5.4	25.1 ± 3.7	0.116
Age (years)	82.3 ± 6.5	81.6 ± 6.7	83.8 ± 5.8	0.254
Arterial hypertension	84	56 (80.0%)	28 (93.3%)	0.138
Diabetes mellitus	20	15 (21.4%)	5 (16.7%)	0.786
Peripheral artery disease	5	5 (7.2%)	0 (0%)	0.318
Stroke/TIA	15	7 (9.1%)	8 (24.2%)	1.000
Pacemaker	3	2 (2.9%)	1 (3.3%)	1.000
Atrial fibrillation	14	9 (12.9%)	5 (16.7%)	0.754
Hemoglobin (g/l)	114.7 ± 17.8	116.9 ± 17.8	109.6 ± 17.0	0.120
COPD	6	6 (8.6%)	0 (0%)	0.174
GFR (Cockcroft Gault; ml/min/1.73 m^2^)	52.0 ± 21.8	53.9 ± 22.6	47.5 ± 19.3	0.218
STS score	4.6 ± 2.9	4.2 ± 2.6	5.2 ± 3.0	0.080
Aortic valve area (cm^2^)	0.60 ± 0.23	0.61 ± 0.22	0.58 ± 0.27	0.420
Mean aortic gradient (mmHg)	41 ± 16	40 ± 17	43 ± 14	0.336
LVEF (%)	57 ± 13	56 ± 13	58 ± 12	0.319
Reduced LVEF < 45%	17	14 (20.0%)	3 (10.0%)	0.262
Total CACS (Median IQR [25–75])	564 [160–1045]	386 [75–765]	1019 [542–1907]	0.001

*BMI* body mass index, *CAD* coronary artery disease, *COPD* chronic obstructive pulmonary disease, *CACS* coronary artery calcium score, *GFR* glomerular filtration rate, *LVEF* left ventricular ejection fraction, *IQR* Interquartile range, *QCA* quantitative coronary angiography, *TIA* transient ischemic attack

### CTA and image quality

In 16 (16%) out of 100 patients, adequate visualization of the entire coronary tree (all 16 segments) was possible with CTA, while 84 (84%) of 100 patients had at least one segment which was not evaluable for CAD analysis due to impaired image quality (e.g. motion artefacts). Out of the 84 patients with non-evaluable segments, a total of 578 segments (578/1533 = 38%) showed a CTA image quality that was insufficient for quantitative CAD evaluation. When focusing only on the left main and proximal segments, 54 out of 100 patients could be assessed, whereas 46% of patients showed at least one segment (i.e. left main or proximal segment) which depicted impaired image quality. On a segment analysis, 301 (75%) out of 400 left main and proximal segments showed good image quality.

### Per patient analysis entire coronary artery tree: the diagnostic performance of CTA and combined CTA/CACS

**Fig. 1:**
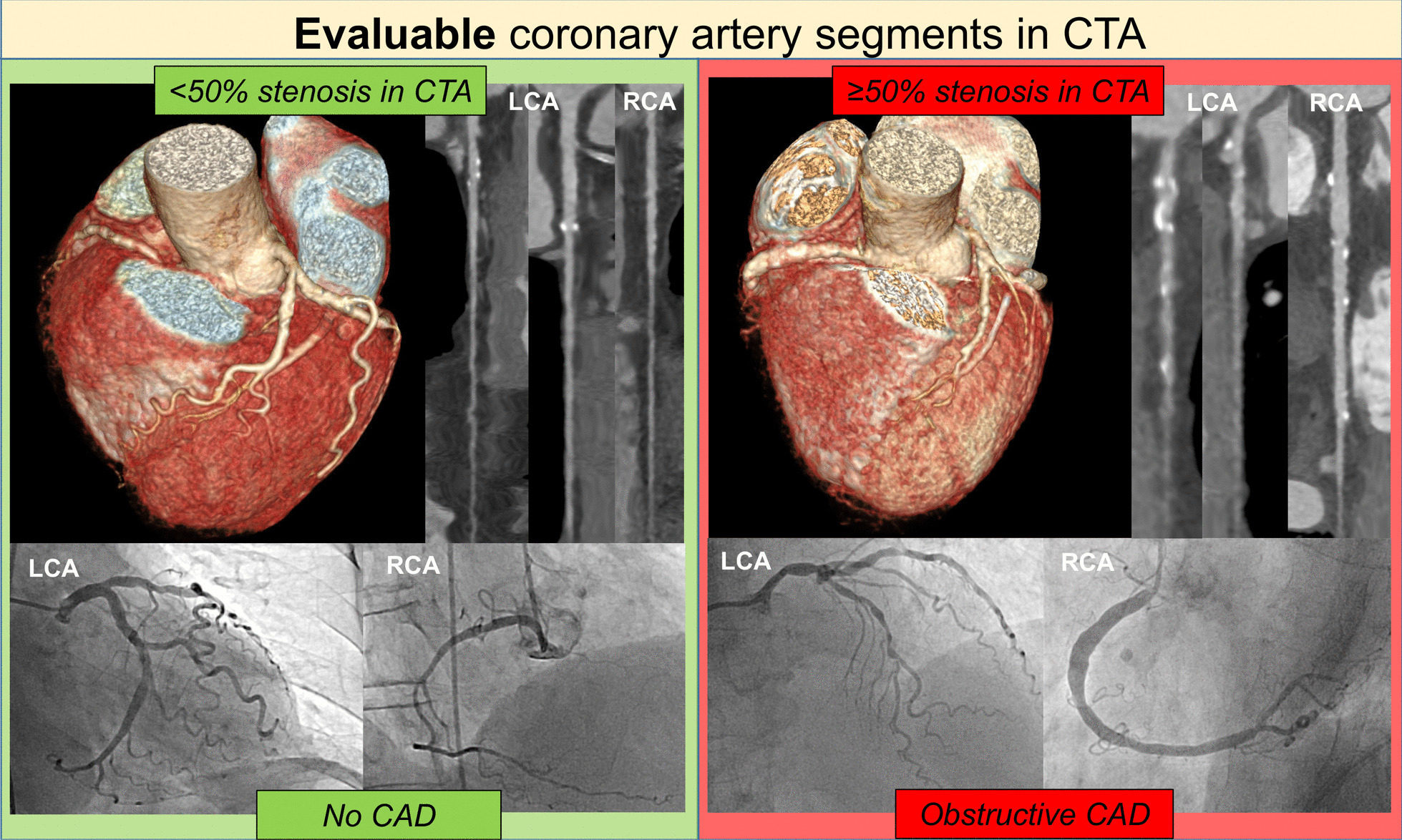
Examples of patients undergoing pre-TAVI CT with “evaluable” coronary artery segments. On the left side a patient is presented where in the pre-TAVI CTA all coronary segments were evaluable. There was no obstructive CAD present in CTA analysis, confirmed by invasive QCA. On the right hand side, a patient is presented, where CTA showed obstructive CAD, confirmed by invasive QCA in the distal left main stem and in the LAD. *CAD* coronary artery disease, *CTA* computed tomography angiography, *RCA* right coronary artery, *LCA* left coronary artery

**Fig. 2:**
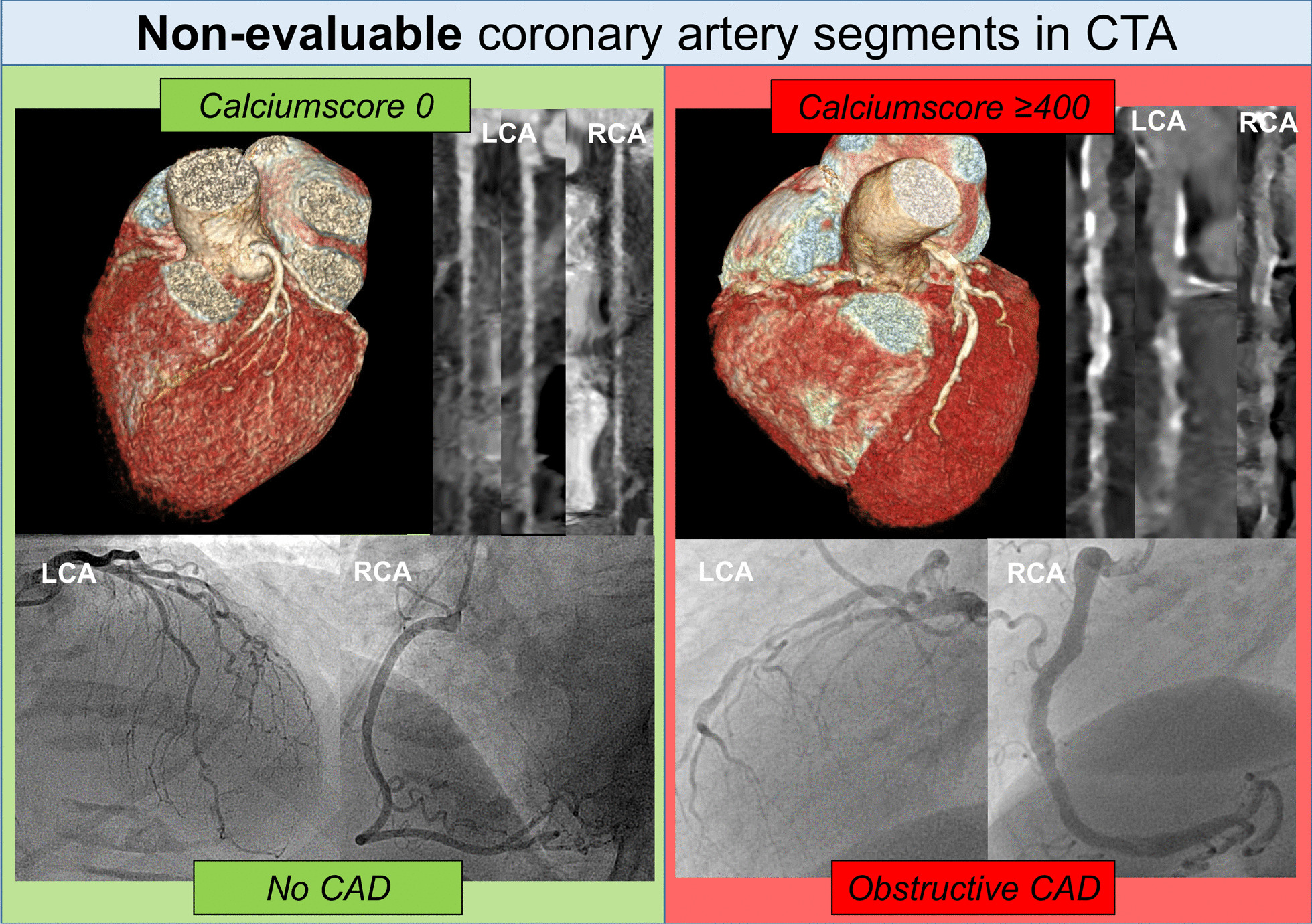
Examples of patients undergoing pre-TAVI CT with “non-evaluable” coronary artery segments and consecutive application of CACS. On the left side a patient is presented where pre-TAVI CTA coronary segments were non- evaluable, however CACS was 0 and no obstructive CAD was confirmed by invasive QCA. On the right hand side, a patient is presented, where CTA was non-evaluable, but CACS was above 400 and invasive QCA confirmed obstructive CAD (i.e. high grade stenosis in the LAD/1.diagnoal branch). *CACS* coronary artery calcium score, *CAD* coronary artery disease, *CTA* computed tomography angiography, *RCA* right coronary artery, *LCA* left coronary artery

The findings of the performance of CTA in diagnosing CAD using 50% diameter stenosis in invasive QCA as a reference are summarized in Table 2 (and for 70% diameter stenosis in Additional file 1: Table S1). On a per-patient analysis, when non-evaluable segments were rated as possible obstructive CAD, CTA showed a sensitivity of 100% (95% CI 88.4–100), specificity of 11.4% (95% CI 5.1–21.3), positive predictive value of 32.6% (95% CI 30.8–34.5), negative predictive value of 100% and diagnostic accuracy of 38% (95% CI 28.5–48.3) for obstructive CAD. On a per-patient analysis excluding patients with non-evaluable segments, (n = 16 included, n = 84 excluded), CTA had a sensitivity of 100% (95% CI 29.2–100), specificity of 61.5% (95% CI 31.6–86.1), positive predictive value 37.5% (95% CI 23.2–54.4), negative predictive value of 100% and diagnostic accuracy of 68.8% (95% CI 41.3–89). On a per-patient analysis including those with non-evaluable segments (n = 100; note that non-evaluable segments are counted as being positive for stenosis) with incorporation of CACS (cut-off ≥ 400), combined CTA/CACS had a sensitivity of 83.3% (95% CI 65.3–94.4), specificity of 50% (95% CI 37.8–62.2), positive predictive value of 41.7% (95% CI 35.0–48.7), negative predictive value of 87.5% (95% CI 75.3–94.2) and diagnostic accuracy of 60% (95% CI 49.7–69.7), see Figs. 1 and 2. Thirty-five percent of the patients were true negative and 5% were false negative. When applying a combined CTA/CACS approach with a CACS cut-off of 100 for patients with non-evaluable segments, the sensitivity and negative predictive value increased to 100% and obstructive CAD could be ruled out in 20% of the patients versus 8%, using CTA alone.

**Table 2:** CTA and combined CTA/CACS versus QCA as the reference standard (≥ 50% stenosis)

	True positive	False positive	False negative	True negative	Sensitivity (%)	Specificity (%)	PPV (%)	NPV (%)	Positive LLR	Negative LLR	Accuracy
*Patient based analysis (total n = 100 patients)*											
Including patients with non-evaluable segments (n = 100 included)	30	62	0	8	100	11.4	32.6	100	1.13	0	38
(95% CI)					88.4–100	5.1–21.3	30.8–34.5	nc	1.04–1.23	nc	28.5–48.3
Excluding patients with non-evaluable segments (n = 16 included, n = 84 excluded)	3	5	0	8	100	61.5	37.5	100	2.60	0	68.8
(95% CI)					29.2–100	31.6–86.1	23.2–54.4	nc	1.31–5.17	nc	41.3–89.0
Combined CTA/CACS (n = 100 included) (If non-evaluable segments present CACS cut-off ≥ 100 = CAD)	30	50	0	20	100	28.6	37.5	100	1.4	0	50
(95% CI)					88.4–100	18.4–40.6	34.1–41.0	nc	1.21–1.62	nc	39.8–60.2
Combined CTA/CACS (n = 100 included) (If non-evaluable segments present CACS cut-off ≥ 400 = CAD)	25	35	5	35	83.3	50.0	41.7	87.5	1.67	0.33	60
(95% CI)					65.3–94.4	37.8–62.2	35.0–48.7	75.3–94.2	1.25–2.21	0.14–0.77	49.7–69.7
*Segment based analysis (all coronary segments, n = 1533 segments)*											
Including non-evaluable segments (n = 1533 included)	41	668	15	809	73.2	54.8	5.8	98.2	1.62	0.49	55.5
(95% CI)					59.7–84.2	52.2–57.3	4.9–6.8	97.2–98.8	1.37–1.92	0.32–0.76	52.9–58.0
Excluding non-evaluable segments (n = 955 included, n = 578 excluded)	19	112	15	809	55.9	87.8	14.5	98.2	4.60	0.50	86.7
(95% CI)					37.9–72.8	85.6–89.9	10.7–19.3	97.4–98.8	3.25–6.49	0.34–0.73	84.4–88.8
*Proximal segment based analysis (left main stem + proximal segment of LAD, LCX, RCA)*											
Including non-evaluable segments (n = 400 included)	15	140	4	241	78.9	63.3	9.7	98.4	2.15	0.33	64.0
(95% CI)					54.4–94.0	58.2–68.1	7.6–12.3	96.2–99.3	1.65–2.81	0.14–0.80	59.1–68.7
Excluding non-evaluable segments (n = 301 included, n = 99 excluded)	8	48	4	241	66.7	83.4	14.3	98.4	4.01	0.40	82.7
(95% CI)					34.9–90.1	78.6–87.5	9.4–21.2	96.4–99.3	2.49–6.46	0.18–0.89	78.0–86.8
*Left coronary artery proximal segment based analysis (left main stem + proximal segment of the LAD)*											
Including all non-evaluable segments (n = 200 included)	3	56	1	140	75.0	71.4	5.1	99.3	2.62	0.35	71.5
(95% CI)					19.4–99.4	64.6–77.6	2.8–9.0	96.2–99.9	1.43–4.82	0.06–1.92	64.7–77.6
Excluding non-evaluable segments (n = 165 included, n = 35 excluded)	3	21	1	140	75.0	87.0	12.5	99.3	5.75	0.29	86.7
(95% CI)					19.4–99.4	80.8–91.7	6.7–22.2	99.29–99.9	2.88–11.49	0.05–1.57	80.5–91.5
*LAD vessel based analysis*											
Including all non- evaluable segments (n = 500 included)	16	207	8	269	66.7	56.5	7.2	97.1	1.53	0.59	57
(95% CI)					44.7–84.4	51.9–61.0	5.4–9.5	95.0–98.4	1.13–2.07	0.33–1.04	52.5–61.4
Excluding non-evaluable segments (n = 338 included, n = 162 excluded)	8	53	8	269	50.0	83.5	13.1	97.1	3.04	0.60	82.0
(95% CI)					24.7–75.4	79.0–87.4	8.0–20.7	95.4–98.2	1.76–5.26	0.37–0.98	77.4–85.9
*LCX vessel based analysis*											
Including non-evaluable segments (n = 500 included)	15	218	4	263	78.9	54.7	6.4	98.5	1.74	0.39	55.6
(95% CI)					54.4–94.0	50.1–59.2	5.1–8.1	96.5–99.4	1.35–2.24	0.16–0.92	51.1–60.0
Excluding non-evaluable segments (n = 299 included, n = 201 excluded)	6	26	4	263	60.0	91.0	18.8	98.5	6.67	0.44	90.0
(95% CI)					26.2–87.8	87.1–94.0	11.0–30.1	96.9–99.3	3.57–12.46	0.21–0.94	86.0–93.1
*RCA vessel based analysis*											
Including all non-evaluable segments (n = 400 included)	10	214	3	173	76.9	44.7	4.5	98.3	1.39	0.52	45.8
(95% CI)					46.2–95.0	39.7–49.8	3.3–6.0	95.5–99.4	1.02–1.90	0.19–1.40	40.8–50.8
Excluding non-evaluable segments (n = 209 included, n = 191 excluded)	5	28	3	173	62.5	86.1	15.2	98.3	4.49	0.44	85.2
(95% CI)					24.5–91.5	80.5–90.5	8.6–25.3	95.9–99.3	2.37–8.49	0.18–1.07	79.6–89.7

*CACS* coronary artery calcium score, *CAD* coronary artery disease, *CI* confidence interval, *CTA* computed tomography angiography, *LAD* left anterior descending artery, *LCX* left circumflex artery, *LLR* likelihood ratio, *nc* not computable/calculable, *NPV* negative predictive value, *PPV* positive predictive value, *QCA* quantitative coronary angiography, *RCA* right coronary artery

### Per patient analysis assessing only left main and proximal segments of LAD, RCA and RCX

When assessing only the left main and proximal segments, 15% showed at least a 50% stenosis in invasive QCA. On a per-patient analysis, when non-evaluable segments were rated as possible obstructive CAD, CTA showed a sensitivity of 80% (95% CI 51.9–95.7), specificity of 29.4% (95% CI 20.0–40.03), positive predictive value of 16.7% (95% CI 13.0–21.1), negative predictive value of 89.3% and diagnostic accuracy of 37% (95% CI 27.6–47.2) for obstructive CAD. On a per-patient analysis excluding patients with non-evaluable segments, (n = 54 included, n = 46 excluded), CTA had a sensitivity of 57.1% (95% CI 18.4–90.1), specificity of 53.2% (95% CI 38.1–68.9), positive predictive value 15.4% (95% CI 8.2–27.0), negative predictive value of 89.3% and diagnostic accuracy of 53.7% (95% CI 39.6–67.4).

### Per segment analysis: the diagnostic performance of CTA

The findings on a per segment analysis for 50% diameter stenosis are depicted in Table 2 (and for 70% stenosis in Additional file 1: Table S1). The per segment analysis, where non-evaluable segments were rated as obstructive CAD, the sensitivity was 73.2% (95% CI 59.7–84.2), specificity 54.8% (95% CI 52.2–57.3), positive predictive value 5.8% (95% CI 4.9–6.8), negative predictive value 98.2% (95% CI 97.2–98.8) and accuracy of 55.5% (95% CI 52.9–58.0).

### Diagnostic performance of CACS versus QCA

**Fig. 3:**
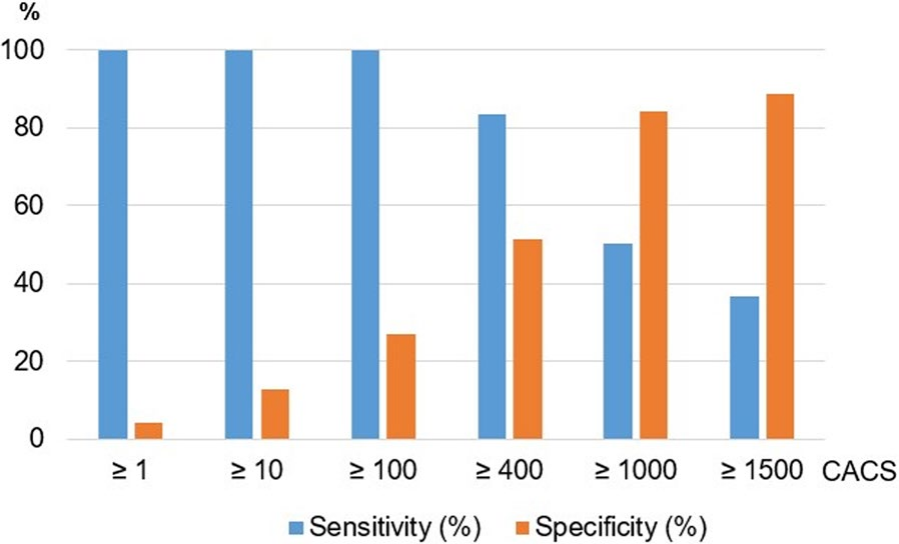
Sensitivity and specificity of CACS in the detection of CAD. In this figure the sensitivity and specificity of different CACS in detecting obstructive CAD (i.e. ≥ 50% stenosis) by invasive QCA as the reference standard is depicted. *CACS* coronary artery calcium score

**Fig. 4:**
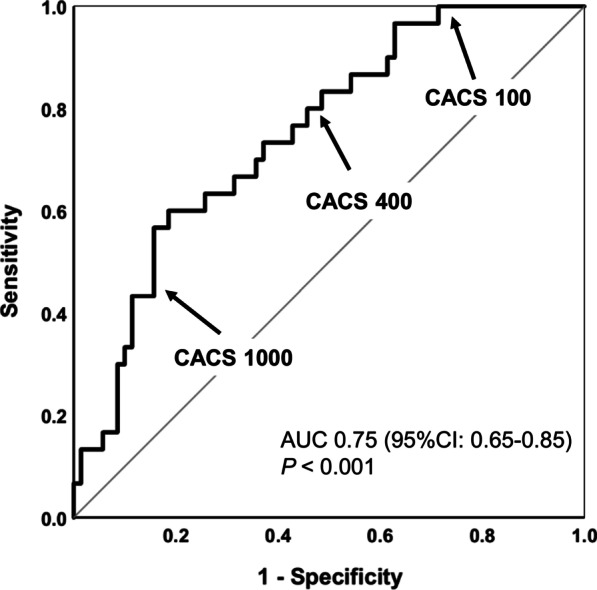
Patient based ROC curve analysis of CACS for the prediction of CAD. Receiver-operator characteristic (ROC) curve identifying the cut-off CACS for prediction of obstructive (50% CAD stenosis in QCA as the reference standard) on a per-patient patient- based analysis. *CACS* coronary artery calcium score

The sensitivities and specificities of different CACS thresholds to diagnose a ≥ 50% diameter stenosis on invasive coronary angiography are shown in Fig. 3. In our cohort, no patient with CACS of less than or equal to 114 was found to have obstructive CAD (≥ 50% stenosis on invasive QCA). Similarly, no patient with a CACS of less than or equal to 187 had ≥ 70% stenosis on invasive coronary angiography. Of the patients who had CACSs ≥ 400, the majority (25/30 or 83%) had at least one ≥ 50% diameter stenosis on QCA while 9/11 (82%) had at least one ≥ 70% diameter stenosis. Figure 4 summarizes the ROC of CACS with different cut-offs in detecting ≥ 50% diameter stenosis by using QCA as the reference standard in patients being assessed for TAVI. An area under the curve of 0.75 was observed with a standard deviation of 0.051 (95% CI 0.651–0.850; *p* < 0.001).

## Discussion

To our knowledge, this is the first blinded study assessing the diagnostic performance of quantitative CTA, CACS and combined quantitative CTA/CACS for CAD in a cohort of consecutive patients undergoing TAVI as compared to the gold standard of quantitative invasive coronary angiography. Our analysis shows that (1) a significant proportion of patients (> 80% of patients) have coronary artery segments that are not evaluable by pre-TAVI CTA where per our institutional protocol no medication for rate control or vasodilatation was used; however, when only focusing on the left main and proximal segments, 56% of the patients and 75% of the segments could be assessed (2) in patients with non-evaluable coronary artery segments, a combined approach using CTA and CACS, resulted in high accuracy for ruling-out CAD (i.e. in 20% of the patients), however with poor diagnostic accuracy. These findings provide evidence that pre-procedural diagnostic workup may be streamlined in selected TAVI patients by potentially omitting routine invasive coronary angiography in selected patients. However, as we have only included patients without prior revascularization or device implants, it has to be noted that these findings are only applicable to these selected patient cohorts. Further, as a high number of TAVI patients show evidence of CAD in CTA or may present with impaired CTA image quality, invasive coronary angiography will still be necessary in a large proportion of patients.

**Fig. 5:**
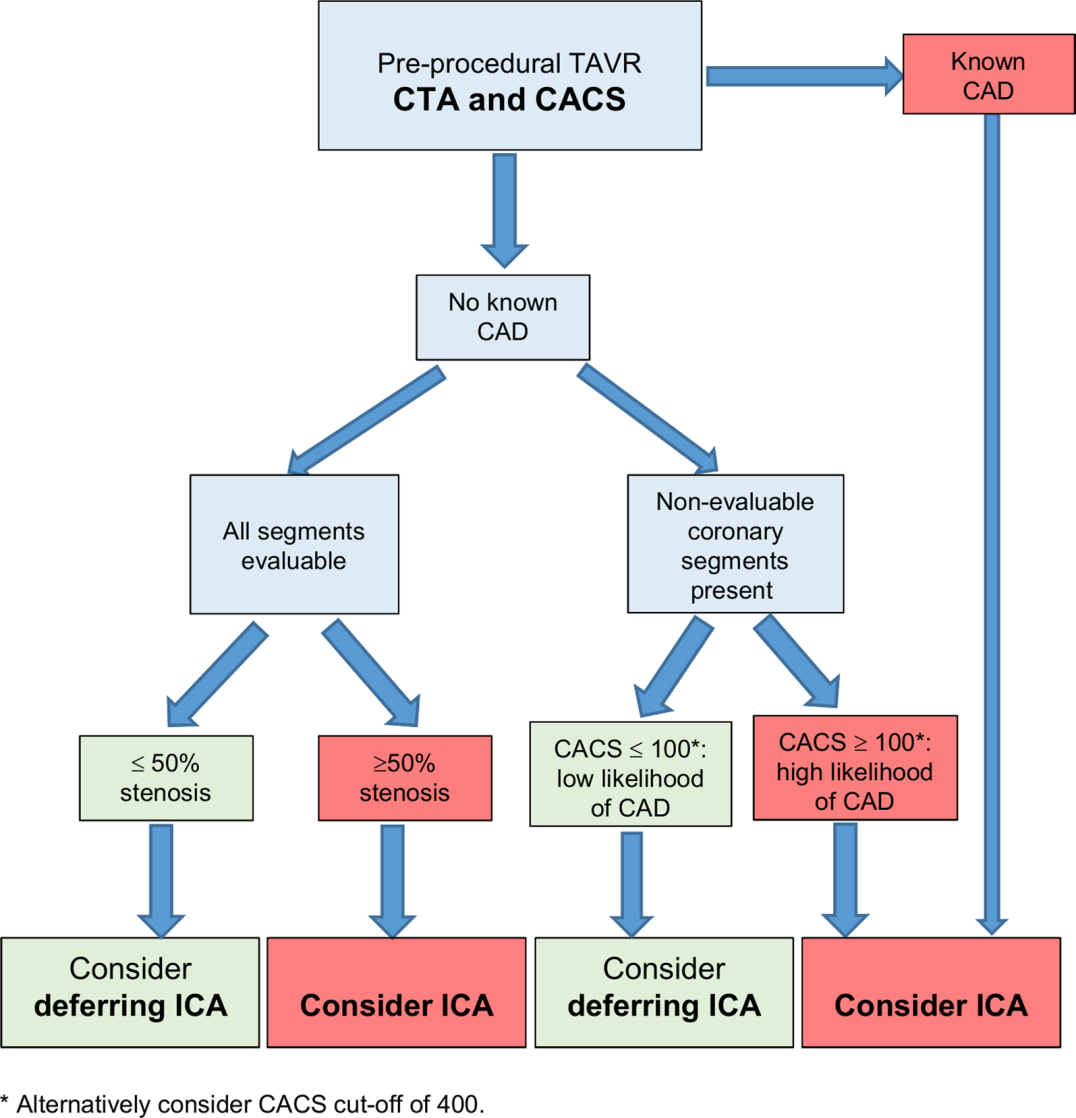
Proposed algorithm for the assessment for CAD in TAVI patients using CTA with CACS. Patients with a known diagnosis of CAD should be considered for ICA to clarify coronary anatomy. In patients with no known CAD and evaluable segments from CTA, the decision to pursue ICA will depend on the presence or absence of a 50% stenosis (left side of figure). In patients with no known CAD and evidence of non-evaluable segments on CTA, CACS thresholds can be incorporated into the decision making process. Decision towards further downstream testing using ICA can be considered in patients with CACS cut-off of 100 with high sensitivity and low specificity (or alternatively 400 with a lower sensitivity and higher specificity) respectively. *CACS* coronary artery calcium score, *CAD* coronary artery disease, *ICA* invasive coronary angiography

Previous investigations have evaluated the pre-TAVI CTA as a diagnostic tool to assess CAD [7, 14, 17, 18, 22, 23]. Similar to our study, in a retrospective analysis of 200 patients with a CAD prevalence of 35.5% (≥ 50% diameter stenosis), CTA without CACS showed a sensitivity and specificity of 100% and 42% respectively and positive and negative predictive values of 48% and 100% respectively. Of note, in this study, the reference standard of invasive CAD was scored by visual assessment as opposed to the validated tool of QCA. Furthermore, the interventional cardiologists who scored the coronary angiograms were not always blinded to the results of CTA. Indeed, when sensitivity analysis was performed, in which the effect of CTA knowledge bias was eliminated, the specificity and positive predictive values were lower. In the current study, coronary angiograms were reviewed without any knowledge of the results from CTA (and vice versa), and the specificity was found to be lower (11.4% vs 42%) with high sensitivity of 100%. A recent review where data were pooled in a patient-level meta-analysis reported similar diagnostic performance compared to our study [23]. In fact, Van den Boogert and colleagues evaluated 7 studies comprising a cumulative sample size of 1275 patients. The per-patient based analysis revealed a pooled sensitivity, specificity, PPV and NPV of 95%, 65%, 71% and 94% respectively. The PPV is somewhat higher than the findings obtained in the current investigation and this may be related to a number of factors. Beta-blocker medications were used in some studies while others did not report whether nitroglycerine was used which may result in increased visualization of the coronary artery tree. Additionally, unblinding effects related to ICA and CTA cannot be ruled out in all studies included. In the current analysis, per our institutional protocol no patient with severe aortic stenosis received heart rate slowing or coronary vasodilating agents, which may both lead to motion artifacts and impaired stenosis quantification. In order to reduce the comparable high reported false positive rates [14, 22, 23], adding CACS might be beneficial in the evaluation of CAD. No patient with a CACS of less than 114 showed obstructive CAD (compared to the ≥ 50% stenosis reference on ICA). The majority of patients with CAD on ICA had a CACS of ≥ 400. When adding CACS to the CTA evaluation in the per patient analysis with non-evaluable segments, the specificity increased from 11 to 29% when using a CACS cut-off of 400, and to 50% when using a CACS cut-off of 100. The true negatives increased from 8 to 20% when using a CACS cut-off of 100 without any false negative patients. The true negative rate could be even increased to 35% when using a CACS cut-off of 400, however with a false positive rate of 5%. CACS thresholds may therefore play an important role when contemplating the added value of CTA in pre-procedural TAVI patients (see proposed flow chart, Fig. 5). This is represented by the fact that when using a combined approach of CTA/CACS, 1/5 of our patient cohort could have safely avoided additional invasive assessment of coronary artery anatomy which may have practical implications as ICA is associated with incremental procedural risk, radiation and risk for contrast induced nephropathy. Furthermore, as the TAVI procedure continues to expand to lower risk patient populations with lower CAD prevalence, assessment of CAD in pre-TAVI CT may streamline the diagnostic work-up and lower cost. A selective approach to ICA in pre-TAVI patients was investigated in a study by Chieffo et al. [7], where 491 patients were evaluated from 2007 to 2013. CTA (without CACS) was used as a first line diagnostic tool and invasive assessment was only performed when coronary segments were not evaluable or if significant CAD was identified on CTA. This approach has been found to be feasible and safe with respect to clinical outcomes.

## Limitations

Although a strength of the current study is the inclusion of a prospective cohort of contemporary patients who underwent TAVI with blinded quantitative CTA/CACS and QCA analysis, the results may not be generalizable to all TAVI patients due the inclusion criteria and low patient number. In fact, as there were quite a large patient number excluded due to prior PCI/stent implantation, coronary artery bypass grafting and previous device implants, the results are only applicable for these selected patients. It remains unclear whether different approaches using CTA alone, combined with CACS or CACS alone using different thresholds according to age, gender and clinical CAD pre-test likelihood are required. Moreover, future studies should aim to investigate these research question in a larger cohort, e.g. in a multi-center trial.

## Conclusions

In routinely acquired pre-TAVI CTA, the image quality was insufficient in a high proportion of patients for the assessment of the entire coronary artery tree. However, when adding CACS in patients with low image quality to quantitative CTA assessment in patients with good image quality, obstructive CAD could be ruled-out in 1/5 of the patients and may therefore constitute a strategy to streamline pre-procedural workup, and reduce risk, radiation and costs in selected TAVI patients without prior coronary revascularization or device implants.

## Supplementary Information

**Additional file 1: Table S1.** CTA/CACS versus QCA as the reference standard (≥ 70% stenosis).

## Data Availability

The datasets used and analyzed during the current study are available from the corresponding author on reasonable request.

## Abbreviations

CACS: Coronary artery calcium score
CAD: Coronary artery disease
CT: Computed tomography
CTA: Computed tomography angiography
ICA: Invasive coronary angiography
LAD: Left anterior descending artery
LCX: Left circumflex artery
LM: Left main
QCA: Quantitative coronary angiography
RCA: Right coronary artery
ROC: Receiver operating characteristics
TAVI: Transcatheter aortic valve implantation
