# Supplementary material for: Diagnostic performance of quantitative coronary artery disease assessment using computed tomography in patients with aortic stenosis undergoing transcatheter aortic-valve implantation
Source: BMC Cardiovasc Disord. 2022 Apr 18;22:178. doi: 10.1186/s12872-022-02623-8 (PMC9014581; doi:10.1186/s12872-022-02623-8)
Supplement: Supplementary file 1 — Additional file 1: Table S1. CTA/CACS versus QCA as the reference standard (≥ 70% stenosis). [file 12872_2022_2623_MOESM1_ESM.docx]

**Table S1. CTA/CACS versus QCA as the reference standard (≥ 70% stenosis)**

|  | **True**  **positive** | **False positive** | **False negative** | **True negative** | **Sensitivity**  **(%)** | **Specificity**  **(%)** | **PPV**  **(%)** | **NPV**  **(%)** | **Positive LLR** | **Negative LLR** | **Accuracy** |
| --- | --- | --- | --- | --- | --- | --- | --- | --- | --- | --- | --- |
|  | | | |  |  |  |  |  |  |  |  |
| **Patient based analysis (total n=100 patients)** | | | |  |  |  |  |  |  |  |  |
| Including patients with non-evaluable segments (n=100 included) | 10 | 75 | 1 | 14 | 90.9 | 15.7 | 11.8 | 93.3 | 1.08 | 0.58 | 24.0 |
| (95% CI) |  |  |  |  | 58.7-99.8 | 8.9-25.0 | 9.8-14.1 | 67.0-99.0 | 0.88-1.33 | 0.08-3.98 | 16.0-33.6 |
| Excluding patients with non-evaluable segments (n=16 included, n=84 excluded) | 0 | 1 | 1 | 14 | nc | 93.3 | nc | 93.3 | nc | 1.07 | 87.5 |
| (95% CI) |  |  |  |  |  | 68.1-99.8 |  | 92.4-94.1 |  | 0.94-1.23 | 61.7-98.5 |
| Combined CTA/CACS (n=100 included)  (If non-evaluable segments present CACS cut-off ≥100) | 10 | 64 | 1 | 25 | 90.9 | 28.1 | 13.5 | 96.2 | 1.26 | 0.32 | 35.0 |
| (95% CI) |  |  |  |  | 58.7-99.8 | 19.1-38.6 | 11.1-16.4 | 78.9-99.4 | 1.01-1.59 | 0.05-2.16 | 25.7-45.2 |
| Combined CTA/CACS (n=100 included)  (If non-evaluable segments present CACS cut-off ≥400) | 7 | 47 | 4 | 42 | 63.6 | 47.2 | 13 | 91.3 | 1.21 | 0.77 | 49.0 |
| (95% CI) |  |  |  |  | 30.8-89.1 | 36.5-58.0 | 8.4-19.5 | 82.3-95.9 | 0.74-1.96 | 0.34-1.74 | 38.9-59.2 |
|  |  |  |  |  |  |  |  |  |  |  |  |
| **Segment based analysis (all coronary segments, n=1533 segments)** | | | | | | | |  |  |  |  |
| Including non-evaluable segments (n=1533 included) | 6 | 622 | 6 | 899 | 50.0 | 59.1 | 1.0 | 99.3 | 1.22 | 0.85 | 59.0 |
| (95% CI) |  |  |  |  | 21.1-78.9 | 56.6-61.6 | 0.5-1.7 | 98.8-99.6 | 0.69-2.16 | 0.48-1.49 | 56.5-61.5 |
| Excluding non-evaluable segments (n=955 included, n= 578 excluded) | 3 | 47 | 6 | 899 | 33.3 | 95.0 | 6.0 | 99.3 | 6.71 | 0.70 | 94.5 |
| (95% CI) |  |  |  |  | 7.5-70.1 | 93.5-96.3 | 2.4-14.4 | 99.0-99.6 | 2.56-17.61 | 0.44-1.11 | 92.8-95.8 |
|  |  |  |  |  |  |  |  |  |  |  |  |
| **Proximal segment based analysis left main stem + proximal segment LAD, LCX, RCA)** | | | | | | | | | | |  |
| Including non- evaluable segments (n=400 included) | 2 | 113 | 0 | 285 | 100 | 71.6 | 1.7 | 100 | 3.52 | 0 | 71.8 |
| (95% CI) |  |  |  |  | 15.8-100 | 66.9-76.0 | 1.5-2.0 | nc | 3.01-4.12 | nc | 67.1-76.1 |
| Excluding non-evaluable segments (n=301 included, n=99 excluded) | 0 | 16 | 0 | 285 | nc | 94.7 | 0 | 100 | nc | nc | nc |
| (95% CI) |  |  |  |  |  | 91.5-96.9 | nc | nc |  |  |  |
|  |  |  |  |  |  |  |  |  |  |  |  |
| **Left coronary artery proximal segment analysis (left main stem + proximal segment of the LAD)** | | | | | | | | |  |  |  |
| Including non-evaluable segments (n=200 included) | 0 | 42 | 0 | 158 | nc | 79.0 | 0 | 100 | nc | nc | nc |
| (95% CI) |  |  |  |  |  | 72.7-84.4 | nc | nc |  |  |  |
| Excluding those with non-evaluable segment (n=165 included, n=35 excluded) | 0 | 7 | 0 | 158 | nc | 95.8 | 0 | 100 | nc | nc | nc |
| (95% CI) |  |  |  |  |  | 91.5-98.3 | nc | nc |  |  |  |
|  |  |  |  |  |  |  |  |  |  |  |  |
| **LAD vessel based analysis** | | |  |  |  |  |  |  |  |  |  |
| Including non-evaluable segments (n=500 included) | 0 | 186 | 4 | 310 | 0 | 62.5 | nc | 98.7 | nc | 1.60 | 62.0 |
| (95% CI) |  |  |  |  | 0-60.2 | 58.1-66.8 |  | 98.6-98.8 |  | 1.49-1.71 | 57.6-66.3 |
| Excluding non-evaluable segments (n=338 included, n=162 excluded) | 0 | 24 | 4 | 310 | 0 | 92.8 | nc | 98.7 | nc | 1.08 | 91.7 |
| (95% CI) |  |  |  |  | 0-60.2 | 89.5-95.3 |  | 98.7-98.8 |  | 1.05-1.11 | 88.3-94.4 |
|  |  |  |  |  |  |  |  |  |  |  |  |
| **LCX vessel based analysis** | | |  |  |  |  |  |  |  |  |  |
| Including non-evaluable segments (n=500 included) | 3 | 209 | 2 | 286 | 60.0 | 57.8 | 1.4 | 99.3 | 1.42 | 0.69 | 57.8 |
| (95% CI) |  |  |  |  | 14.7-94.7 | 53.3-62.2 | 0.7-2.9 | 98.0-99.8 | 0.69-2.93 | 0.24-2.03 | 53.3-62.2 |
| Excluding non-evaluable segments (n=299 included, n=201 excluded) | 2 | 9 | 2 | 286 | 50.0 | 97.0 | 18.2 | 99.3 | 16.39 | 0.52 | 96.3 |
| (95% CI) |  |  |  |  | 6.8-93.2 | 94.3-98.6 | 6.4-41.8 | 98.2-99.7 | 5.08-52.92 | 0.19-1.37 | 93.5-98.2 |
| **RCA vessel based analysis** | | |  |  |  |  |  |  |  |  |  |
| Including non-evaluable segments (n=400 included) | 3 | 201 | 0 | 196 | 100 | 49.4 | 1.5 | 100 | 1.98 | 0 | 49.8 |
| (95% CI) |  |  |  |  | 29.2-100 | 44.4-54.4 | 1.3-1.6 | nc | 1.79-2.18 | nc | 44.7-54.8 |
| Excluding non-evaluable segments (n=209 included, n=191 excluded) | 1 | 12 | 0 | 196 | 100 | 94.2 | 7.7 | 100 | 17.33 | 0 | 94.3 |
| (95% CI) |  |  |  |  | 2.5-100 | 90.2-97.0 | 4.6-12.6 | nc | 10.01-30.02 | nc | 90.2-97.0 |

CACS = Coronary artery calcium score; CAD = Coronary artery disease; CI = Confidence interval; CTA = Computed tomography angiography; LAD = Left anterior descending artery; LCX = Left circumflex artery; LLR = Likelihood ratio; nc = not computable/calculable; NPV = Negative predictive value; PPV = Positive predictive value; QCA = Quantitative coronary angiography; RCA = Right coronary artery
